# New approaches to measles eradication, with special reference to Africa

**DOI:** 10.11604/pamj.supp.2025.51.1.47547

**Published:** 2025-06-13

**Authors:** William Mbabazi, Robert Davis

**Affiliations:** 1 World Health Organization, Juba, South Sudan,; 2 Muthaiga Heights, Nairobi, Kenya

**Keywords:** Immunization, measles, eradication, Africa

## Abstract

Measles eradication, not yet the target of a global resolution, awaits global commitments by governments and partners. This essay examines ten proposed innovations that aim to bring the global community closer to the date when it will be prepared to launch a global eradication effort. We look especially at the value of synergies between measles elimination and Global Polio Eradication Initiative (GPEI), the Global Polio Eradication Initiative. The underfunded GPEI could be re-energized by closer integration with measles/rubella eradication. This point is especially relevant in an era of declining donor commitment to public health. Above all, we are pushing for new approaches more quickly to reach eradication of measles with approaches that complement the goals of polio eradication.

## Essay

In 2024, the world celebrated the fiftieth anniversary of the Expanded Programme on Immunization. Soon after the founding of Expanded Programme on Immunization (EPI), there were calls, in 1982, for the global eradication of measles, reinforced by a call from Kofi Annan for a political commitment to global eradication. Those calls, not yet implemented on a global scale, have led to national and regional efforts to eliminate measles transmission in countries and regions. The Region of the Americas has made the most progress towards elimination. In recent years, the Region of the Western Pacific has made great strides towards elimination in its member States, notably China, with the world´s largest population.

An overview of worldwide progress since the turn of the century [1] noted that measles vaccination saved 60 million lives during the period 2000-2023. During 2001-2019, estimated coverage with MCV1 (first dose of measles-containing vaccine) increased worldwide from 71 percent to 86 percent, then declined to 81 percent in 2021 during the COVID-19 pandemic, rising to 83 percent in 2022 and 2023. It is only in the high-income countries that measles measles-containing vaccine (MCV) routine coverage has achieved levels consistent with measles elimination. In low- and middle-income countries, a combination of routine immunization and supplementary immunization activities (SIAs) can interrupt transmission. Where routine (RI) and SIA coverage is suboptimal, transmission continues, sometimes with nationwide epidemics. The African Region of WHO., with its 47 countries, presents a wide spectrum of vaccination performance. Although several countries, especially in southern Africa, have made much progress towards measles elimination [2], the region is also home to three countries (the Democratic Republic of the Congo, Ethiopia, and Nigeria) with problems of intracountry disparities, access issues, and weak infrastructures.

The WHO/UNICEF estimates of MCV1 and MCV2 coverage for 2023 in the Africa Region were 69 and 45 percent, respectively. The reasons for MCV2 underperformance require further research [3]. With weak routine vaccination, 24 of 47 countries did MCV SIAs in 2022-2023, with 144 million children vaccinated. However, only half the campaigns reached 95 percent coverage. The single most important impediment to stopping measles transmission in Africa is the quality of MCV supplementary immunization activities (SIAs) in countries that have poor MCV2 coverage. No country in the region had attained verification criteria for measles elimination by the end of 2023 [4]. Most SIAs in Africa are financed by the GAVI Vaccine Alliance, which has a slow turnaround time of approximately 18 months from initial contact to SIA implementation. This does not always permit the timely implementation of SIAs at the most suitable dates on epidemiological grounds.

Despite progress, the current decade has seen a number of challenges to measles eradication. The Global Polio Eradication Initiative has not yet stopped persistent transmission of wild poliovirus in the two remaining endemic countries, Afghanistan and Pakistan, and transmission of vaccine-derived poliovirus continues in dozens of countries, especially African countries, not endemic for WPV. The global response to the COVID-19 pandemic, necessary as it was, has reduced financial and other resources available for other public health activities. The pandemic and post-pandemic world saw global declines in all routine immunization antigens, including measles. The large cohorts of unvaccinated children carried through the pandemic response have not all been reached due to uneven implementation of the big catch-up campaigns. Poverty and inequity remain persistent challenges, especially in low-income countries with inter- and intra-country variations in MCV coverage. Conflict situations reduce access, often requiring either mass campaigns or PIRI (periodic intensification of routine immunization). These challenges notwithstanding, recent years have seen innovations in the following areas:

### Maintenance, in the Americas, of freedom from endemic measles

Among the six WHO regions, the Region of the Americas is the only one to have verified measles elimination (2016). The Region´s Verification Commission was disbanded once verification was achieved, but reconstituted after the reappearance of endemic measles in Brazil and Venezuela. Sustaining efforts is essential because the measles vaccine saves more lives than any other vaccine. Measles vaccination performance remains an indicator of national capacity to maintain health security and a timely response to future infectious disease threats [5]. De Quadros and colleagues have pointed out that maintenance of regional elimination in the Region of the Americas makes global eradication look far more feasible [6]. So long as the western hemisphere remains free of endemic measles, the skeptics of global eradication will have a weak case to defend.

### Microarray patch vaccination for measles and measles rubella (MR) vaccines

Encouraging field results of MR-MAPs from the Gambia [7,8] have led to published discussions on how best to use MCV patch vaccines on a large scale. Several WHO authors have proposed the following uses for such vaccines: i) delivery at fixed health posts, ii) delivery through outreach sessions conducted by health workers, iii) administration by community health workers (CHWs) [9]. The possibility of CHWs giving measles vaccines house to house during SIAs brings with it the potential for raising SIA coverage through the house-to-house strategy long employed in polio SIAs. This is especially important in Africa, where weaknesses in routine immunization mean that very high-quality SIAs are essential for stopping transmission. Since the MR-MAP is not reconstituted, use of MR-MAPs eliminates the rare cases of erroneous mixing of vaccine with contaminated diluent.

Although MR-MAPs have not yet been used on a national scale, a 2024 analysis suggests that their broad deployment can potentially reach an additional 80 million children between 2030 and 2040, thereby averting 400,000 measles deaths [10]. Richardson and Moss have discussed in detail the hurdles to be overcome in licensing and prequalification of MR-MAPs for measles and rubella [11]. Once patch vaccines become widely available, there will likely be the introduction of house-to-house MCV SIA, using microarray patch vaccines. Such SIAs would have the potential to quickly raise MCV coverage in low- and middle-income countries of Africa, where persistent measles transmission has heretofore impeded progress towards regional measles elimination. Resizing of cold chain facilities is a sine qua non for the successful introduction of microarray patch vaccines at the country level. The Global Polio Eradication Initiative has, since the year 2000, implemented house-to-house SIA vaccination with the oral polio vaccine (OPV). Since OPV is a needle-free technology, it can be given by laypersons with no knowledge of injection technologies. Needle-free technology for MCV administration holds forth the promise of administration by laypersons, such as community health workers, especially in MCV campaigns.

### Use of five-dose vials for measles and measles rubella vaccines

The Republic of Zambia has documented the benefits of adopting 5-dose vials of MCV in a country that previously used 10-dose vials [12]. In Zambia, as elsewhere, the fear of excessive vaccine wastage has heretofore discouraged health workers from opening a 10-dose vial to vaccinate the few children who present for vaccination, especially in small clinics. Districts that used the 5-dose MR saw increases in coverage and reduced vaccine wastage. Health workers were more willing to open a 5-dose vial. India´s decision to switch over from 10- to 5-dose vials has created a larger market for 5-dose vials. WHO and UNICEF have published guidance to countries seeking to transition to 5-dose vials [13].

### New approaches to routine immunization, especially targeting zero-dose children

The disease burden of measles and other vaccine-preventable diseases will be reduced to the extent that health systems succeed in reaching children who have received no doses of any vaccine (zero-dose children). The vaccine alliance, GAVI, has committed $500 million for the period 2021-2025 [14] to reach zero-dose children, two-thirds of whom live in five countries: Nigeria, India, the Democratic Republic of the Congo, Pakistan, and Ethiopia. Approaches to zero-dose children vary according to local conditions. In some countries, zero-dose children are concentrated in remote rural areas. In others, such as northern Nigeria and parts of the DRC, zero-dose children are concentrated in conflict areas with access issues [15]. Wherever gender inequities persist, these must be tackled as part of an approach to reaching zero-dose children. The American Red Cross and partners are undertaking one approach to reaching zero-dose children, notably in Kenya [16] and Zambia. Red Cross volunteers do community mapping of unvaccinated and under-vaccinated children and collaborate with health authorities to ensure their vaccination. This “Five Point Plan” is then independently evaluated to quantify coverage improvements.

### Improvements in immunization information management systems

When EPI was launched in 1974, most countries relied on vaccination registers filled up in and with ink. Monthly summaries were sent to the district, provincial, and national levels, with copying errors possible at every level. While many countries continue to use legacy systems, there is a growing trend towards the use of electronic immunization registers, typically using the internationally adopted DHIS2 package [17], developed by the University of Oslo and partners, which permits timely data analysis for action. DHIS2 permits bottleneck analysis where performance is suboptimal, the generation of user-friendly visuals, and timely tracking of adverse events following immunization (AEFI). DHIS2 has enabled, notably, the monitoring of the MR vaccination of 35 million children in Bangladesh and the tracking of an integrated MR/polio campaign in Uganda, which reached 18 million children. None of these activities are new, but legacy systems did not enable timely data analysis and the taking of corrective measures. The next logical step will be to link health facility vaccination records to health workers´ telephones, so that the child coming in at 6 weeks of age for the first vaccination visit is the subject of SMS messages to the mother for subsequent visits. Reminder systems for mothers and other caregivers have had an excellent track record in reducing dropout rates, as confirmed by historical Cochrane reviews [18]. A recent review of 25 studies confirms the view that text message reminders have a positive impact on vaccination uptake [19].

### Universal adoption of two-dose regimes, with improvements in MCV2 coverage in the second year of life

Since one dose of the measles vaccine rarely achieves 95 percent efficacy, vaccination of all susceptible children with a single dose is unlikely to stop transmission. In the present century, there has been a growing trend towards vaccinations in the second year of life, for measles and other antigens. The 2YL approach is essential for MCV and, increasingly, in countries giving the malaria vaccine. The MCV2 visit can also serve as an opportunity to give vitamin A and anthelminthic drugs. During the period 2000-2019, MCV2 coverage increased dramatically. In the African Region of the WHO, MCV2 coverage rose from 7 to 49 percent from 2013 to 2023. By the end of 2023, 43 of the 47 member states of WHO/AFRO had adopted the two-dose regime [20].

### Wide age range SIAs targeting under-15 populations

The majority of MCV SIAs target those aged 9 to 59 months. Such campaigns target most but not all susceptible. Since many older children have received neither vaccination nor natural exposure to the measles virus, a wide age range campaign, when affordable, protects more children and has a better chance of interrupting transmission. A study of a wide age range of SIAs in the African region found that confirmed measles incidence dropped significantly in 13 of the 17 countries studied [21].

### Integrated SIAs

For reasons not purely technical, many countries continue to implement stand-alone campaigns for single diseases. This writer recently witnessed, in one African capital, three consecutive vaccination campaigns, one each for measles, yellow fever, and cholera, in a single month. Especially in resource-scarce settings, integrated SIA is a logical choice. Nigeria has shown the economic and operational advantages of integrated multi-antigen campaigns, which minimize the time demands on mothers and children, in addition to spreading supervisory and other costs over several antigens [22].

### Advocacy based on economic approaches

Faced with coverage discrepancies among poor, middle-income income and wealthy countries, donors may well ask themselves why they should put additional resources into global measles eradication, an expensive multi-year effort. One answer, quite aside from humanitarian considerations, is the argument from enlightened self-interest. The US was among the principal donors to the global Smallpox Eradication Programme. With smallpox now eradicated, the US saves the total of all its contributions every 26 days because it does not have to vaccinate or treat the disease [23]. As with smallpox, so with measles. Global figures on benefits from MCV vaccination are hard to come by. Kimberly Thompson´s estimates for the United States show historical and projected prevention of 228,000 measles deaths, with $310 billion in averted costs from measles cases, even without incorporating avoided productivity losses and intangible costs [24]. Such analyses need to be done on a global scale as an advocacy tool for global measles or measles/rubella eradication, comparing the costs of a time-limited global campaign to those of long-term control. It should be borne in mind that in any benefit/cost analysis of time-limited eradication, the numerator extends indefinitely into the future, whereas the denominator is time-limited. Hence, one is likely to expect a favorable benefit/cost ratio from the economic analysis of disease eradication programmes.

### Rapid diagnostic tests for global measles surveillance

Point-of-use tests would permit faster turnaround times for confirmation of suspected measles and rubella cases, and, in case of outbreaks, enable faster turnaround times for epidemic response. Until prompt response to reported measles outbreaks is standard operating procedure, endemic countries will continue to pay a heavy price to measles in avoidable morbidity and mortality [25].

**Table 1:** measles vaccination coverage, 2023, by income level

Low-income countries	Middle-income countries	High-income countries
64%	86%	94%

## Conclusion

Efforts to quantify the effect of donor funding on vaccine coverage have shown a significant positive impact of aid, especially with new vaccines [26]. The corollary to this finding is that a reduction in donor funding, notably GAVI funding, may endanger gains made during the golden age of abundant donor support. All parties, especially donors, need to know that external as well as internal financing is essential to any global eradication initiative. The management apparatus of a future global eradication initiative is a key element to its success. In their discussion of elimination, Goodson and colleagues have proposed a diagonal approach, combining the advantages of vertical and integrated approaches [27]. They point out the advantages of using the resources of the GPEI for measles/rubella eradication. GPEI can serve as a prequel to measles eradication. The flagging, underfunded GPEI could be re-energized by closer integration with measles/rubella eradication. This point is especially relevant in an era of declining donor commitment to public health. Winter and Moss, in their discussion of paths to measles eradication, have pointed to the advantage of going big and going fast [28]. Concentration of resources in all countries for less than a decade is preferable to low or medium level resource commitments over an extended period of time. This means firm political and financial commitments unwaveringly on the prize. Polio eradication has been a marathon. Measles eradication should be a sprint.
